# *Ex vivo* multiplex profiling of protein tyrosine kinase activities in early stages of human lung adenocarcinoma

**DOI:** 10.18632/oncotarget.19803

**Published:** 2017-08-02

**Authors:** Stephan Arni, Thi Hong Nhung Le, Rik de Wijn, Refugio Garcia-Villegas, Martjin Dankers, Walter Weder, Sven Hillinger

**Affiliations:** ^1^ Department of Thoracic Surgery, University Hospital Zürich, Zürich, Switzerland; ^2^ PamGene International B.V., ‘s-Hertogenbosch, The Netherlands; ^3^ Department of Physiology, Biophysics and Neuroscience, Centro de Investigación y de Estudios Avanzados del IPN, Mexico City, Mexico

**Keywords:** protein tyrosine kinase, lung adenocarcinoma, protein activity microarrays, molecular markers of metastasis and progression, methodology for proteomics

## Abstract

Despite constant improvement in existing therapeutic efforts, the overall survival rate of lung cancer patients remains low. Enzyme activities may identify new therapeutically targetable biomarkers and overcome the marked lack of correlation between cellular abundance of translated proteins and corresponding mRNA expression levels. We analysed tyrosine kinase activities to classify lung adenocarcinoma (LuAdCa) resection specimens based on their underlying changes in cellular processes and pathways that are agents of or result from malignant transformation. We characterised 71 same-patient pairs of early-stage LuAdCa and non-neoplastic LuAdCa resection specimen lysates in the presence or absence of a tyrosine kinase inhibitor. We performed *ex vivo* multiplex tyrosine phosphorylation assays using 144 selected microarrayed kinase substrates. The obtained 76 selected phosphotyrosine signature peptides were subsequently analysed in terms of follow-up treatments and outcomes recorded in the patient files. For tumour, node, metastasis (TNM) stage 1 LuAdCa patients, we noticed a larger tyrosine kinase inhibitor-induced decrease in tyrosine phosphorylation for long-term as opposed to short-term disease survivors, for which 26 of 76 selected peptides were significantly (*p < 0.01*, FDR < 3%) more inhibited in the long-term survivors. Using statistical class prediction analysis, we obtained a 'prognostic-signature' for long- versus short-term disease survivors and correctly predicted the survival status of 73% of our patients. Our translational approach may assist clinical disease management after surgical resection and may help to direct patients for an optimal treatment strategy.

## INTRODUCTION

Adenocarcinoma, squamous cell carcinoma and large cell carcinoma represent the main subtypes of non-small cell lung cancer (NSCLC), the leading cause of lung cancer-related deaths [1]. At present, the extent of NSCLC is assessed using the tumour, node, metastasis (TNM) staging system, based on anatomical criteria including tumour size, lymph node status and awareness of the presence of metastasis [2]. The TNM staging system assists clinicians in evaluating a prognosis and formulating treatment modalities [2]. Unfortunately, even with existing therapeutic efforts, the 5-year relative survival rate varies markedly depending on the stage at diagnosis, from 49% to 16% to 2% for patients with local, regional, and distant-stage disease, respectively [3]. For lung cancer patients with regional- or distant-stage diseases, the poor prognosis reflects the lack of therapeutic options available to treat this disease. Smoking prevention and cessation programmes have been implemented and new screening efforts such as low-dose helical computed tomography are proposed to increase the detection rate of high-risk patients or patients with local disease, respectively [4–6]. Nonetheless, even with an improved detection rate of local-stage disease amenable to surgery, the predicted and real outcomes vary substantially with similarly stratified lung cancer status. To improve the discriminative power of the TNM staging system both in terms of reliability and accuracy at sub-classifying high- versus low-risk patients, a thorough search for new prognostic and predictive molecular biomarkers is of utmost importance. For TNM stage 1 NSCLC patients, surgery is the current standard of care, but guidelines also suggest a benefit of adjuvant chemotherapy in some high-risks patients with primary tumour ≥4 cm [7]. After surgery, biomarkers that molecularly characterise stage 1 lung adenocarcinoma (LuAdCa) resection specimens may identify high–risk patients for either adjuvant chemotherapy or radiotherapy [8], and reciprocally may select for low-risk patients who qualify to safely avoid adjuvant therapies.

Despite tremendous efforts, the search for such reliable lung cancer-specific molecular biomarkers for local stage NSCLC has remained unsuccessful, which may be attributed to a lack of correlation between mRNA expression levels and the cellular abundance of their corresponding protein [9, 10]. Moreover, protein- and gene-expression profiling methods may fail to detect important modulations of enzyme activities caused either by posttranslational events during tumour progression and/or treatment response [11, 12]. To circumvent these limitations, we have developed Activity-Based Protein discovery platforms with LuAdCa resection specimens to search and identify enzymatic activities as prognostic and predictive biomarkers [13–15].

In both eukaryotic and prokaryotic biological systems, protein phosphorylation is a ubiquitous and reversible post-translational modification. More than 500 protein kinase-coding motifs have been identified in humans [16]. Ninety are unique tyrosine kinase genes [17] and, as key regulators of cell functions, are directly involved in numerous signal transduction cascades [18]. Related protein kinases may have many common substrates, and subtle differences in protein kinase activities may determine, *in vivo*, the triggering of relevant signalling pathway [19]. Since the discovery of high tyrosine kinase activities in cancer [20], pharmaceutical companies have been conducting intense research to develop highly selective protein tyrosine kinase inhibitors (PTKI). PTKI such as erlotinib (Tarceva) or gefitinib (Iressa) are prescribed for subsets of NSCLC patients with regional stage disease and with enhanced tumour tyrosine kinase activities, but they are not the primary approved standard of care for early stage LuAdCa patients [21]. PTKI provide only temporary relief for molecularly unselected NSCLC patients. This high rate of treatment failure [22] is due both to the high plasticity of lung cancer to develop secondary resistance [23] and to an incomplete understanding of the molecular mechanisms involved in lung carcinogenesis.

By employing a same-patient set of lung resection specimens, we measured the tyrosine kinase activities in non-neoplastic and adenocarcinoma lysates, in addition to adenocarcinoma lysates containing gefitinib with a multiplex profiling approach using well-characterised tyrosine kinase substrates [24]. The gefitinib added to the tumour lysates was not intended to select for patients who may be sensitive to this PTKI. In this exploratory study, gefitinib was used as an assay tool applied in combination with clinical follow-up to test the discriminative power of our molecular signature for low-risk versus high-risk patients with respect to treatment response and survival. This molecular prognosis signature based on tyrosine kinase activity differences found in LuAdCa resection specimens may also lead to the identification of novel targets for future anti-lung cancer therapies.

## RESULTS

### Patient characteristics

We have summarized the clinical parameters of 49 TNM stage 1 and 22 TNM stage 2 LuAdCa patients enrolled in this study (Table 1, groups A to H). Supplementary Table 1 provides the detailed clinical characteristics of the patients. The TNM stage 1 training cohort contained 10 short-term survivors with a median disease-specific survival (DSS) of 34.6 months (group A, range 10.4-52.9 months) and 10 long-term survivors with a median patient DSS of 73.9 months (group B, range 62.9-99.3 months). The TNM stage 1 validation cohort contained 3 short- and 14 long-term disease-specific survivors including two patients receiving adjuvant chemotherapy. The median DSS time for the validation cohort was 59.6 months (group C, range 11.7-99.2 months). To evaluate the class prediction model for the TNM stage 1 signature, we omitted 12 patients from the validation cohort that were still alive at the time of the analysis but with a follow-up time shorter than the cut-off time set at 53.6 months of survival (see calculation of cut-off time in Material and Methods, group D, range 0.7-42.2 months).

**Table 1:** Patient characteristics

	TNM 1 short term survivors trainingset Group A (n=10)	TNM 1 long term survivors trainingset Group B (n=10)	TNM 1validatingset Group C (n=17)	TNM 1short follow upsetGroup D (n=12)	TNM 2 short term survivors training set Group E (n=5)	TNM 2 long term survivors training set Group F (n=5)	TNM 2validatingset Group G (n=7)	TNM 2 short follow upset Group H (n=5)
Gender								
Male n(%)	6 (60)	5 (50)	8 (47)	1 (8)	2 (40)	2 (40)	4 (57)	2 (40)
Female n(%)	4 (40)	5 (50)	9 (53)	11 (92)	3 (60)	3 (60)	3 (43)	3 (60)
Age								
Median years at surgery (range)	65.5 (46-74)	64 (54-75)	64 (28-80)	64.5 (41-84)	67 (52-70)	80 (48-88)	65 (56-76)	61 (49-68)
Median months survival (range)	34.6 (10.4-52.9)	73.9 (62.9-99.3)	59.6 (11.7-99.2)	24.3 (0.7-42.2)	12.2 (8.1-23.3)	36.9 (30.6-73.5)	29.6 (23.3-98.2)	19.1 (1.3-23.5)
TNM								
Stage 1A n(%)	4 (40)	5 (50)	5 (29)	3 (25)	0 (0)	0 (0)	0 (0)	0 (0)
Stage 1B n(%)	6 (60)	5 (50)	12 (71)	9 (75)	0 (0)	0 (0)	0 (0)	0 (0)
Stage 2A n(%)	0 (0)	0 (0)	0 (0)	0 (0)	2 (40)	2 (40)	1 (16)	3 (60)
Stage 2B n(%)	0 (0)	0 (0)	0 (0)	0 (0)	3 (60)	3 (60)	6 (84)	2 (40)
N0 n(%)	10 (100)	10 (100)	17 (100)	12 (100)	2 (40)	3 (60)	4 (57)	2 (40)
N1 n(%)	0 (0)	0 (0)	0 (0)	0 (0)	3 (60)	2 (40)	3 (43)	3 (60)
Tobacco pack-years								
None n(%)	0 (0)	0 (0)	3 (17.6)	1 (8.3)	4 (80)	1 (20)	1 (15)	2 (40)
<30 n(%)	1 (10)	2 (20)	5 (29.5)	3 (25)	0 (0)	2 (40)	4 (55)	1 (20)
31-49 n(%)	3 (30)	5 (50)	3 (17.6)	0 (0)	0 (0)	2 (40)	1 (15)	1 (20)
>50 n(%)	6 (60)	3 (30)	6 (35.3)	8 (66.6)	1 (20)	0 (0)	1 (15)	1 (20)
EGFR status								
Mutated EGFR mutated/tested (% mutated)	0/5 (0)	0/2 (0)	2/8 (25)	0/10 (0)	1/4 (25)	0/2 (0)	0/3 (0)	0/3 (0)
Amplified EGFR amplified/tested (% amplified)	1/5(20)	0/2 (0)	2/8 (25)	3/9 (33)	3/4 (75)	1/2 (50)	0/2 (0)	1/3 (30)
Mutated or amplified EGFR positive/tested (% positive)	1/5 (20)	0/2 (0)	3/8 (37.5)	3/9 (33)	3/4 (75)	1/2 (50)	0/3 (0)	1/3 (30)
Disease status								
Locally recurrent (yes/no/unknown)	(8/1/1)	(0/10/0)	(7/10/0)	(0/12/0)	(4/1/0)	(2/3/0)	(0/7/0)	(0/5/0)
Metastatic (yes/no/unknown)	(8/1/1)	(0/10/0)	(4/11/2)	(0/12/0)	(5/0/0)	(2/3/0)	(1/6/0)	(0/5/0)
Adjuvant therapy n(%)	0 (0)	0 (0)	2 (12)	2 (17)	2 (40)	1 (20)	4 (57)	3 (60)
Cause of death								
Alive n(%)	0 (0)	10 (100)	11 (65)	12 (100)	0 (0)	5 (100))	6 (85)	5 (100)
Deceased of Lung cancer n(%)	10 (100)	0 (0)	6 (35)	0 (0)	5 (100)	0 (0)	1 (15)	0 (0)

The TNM stage 2 LuAdCa training cohort contained either 5 short-term survivors with a median DSS of 12.2 months (group E, range 8.1-23.3 months) or 5 long-term survivors with a median DSS of 36.9 months (group F, range 30.6-73.5 months). The TNM stage 2 validation cohort included 6 short- and 1 long-term disease-specific survivors with a median DSS of 29.6 months (group G, range 23.3-98.2 months). To evaluate the class prediction model for TNM stage 2, we omitted 5 patients from the validation cohort that were still alive at the time of the analysis but with a follow up time shorter than the cut-off time set at 24 months of survival (group H, range 1.3-23.5 months).

### Kinomes of long- versus short-term TNM stage 1 LuAdCA survivors were maximally discriminated when gefitinib was present during *ex vivo* tyrosine phosphorylation assay

**Figure 1:**
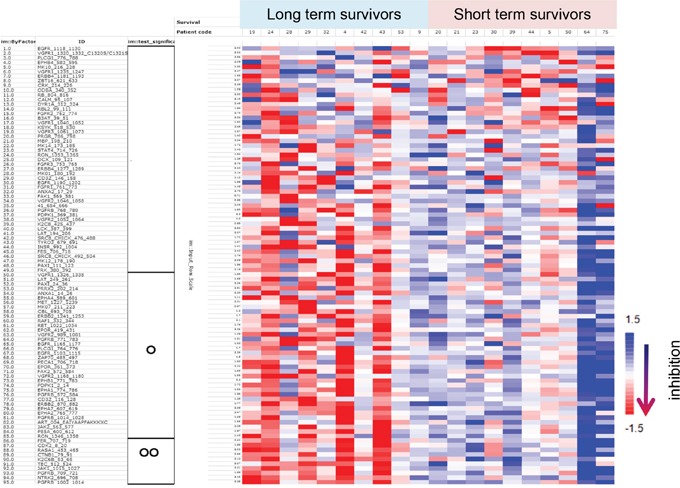
Tyrosine phosphorylation of 95 selected peptide substrates in the presence of gefitinib The 95 PCA-selected peptides are represented as “_inh_S” values, a Log2-transformed ratio of tyrosine phosphorylation. TNM stage 1 LuAdCa patients are plotted in an inhibitory heatmap according to either long- or short-term survival status. Inhibition is scaled per peptide and red colour indicates greater inhibition of phosphorylation. The significance obtained in a per peptide t-test is indicated on the left side of the figure using the following coding scheme: OO, *p* < 0.01; O, *p* < 0.05; *p* ≥ 0.05 otherwise.

**Figure 2:**
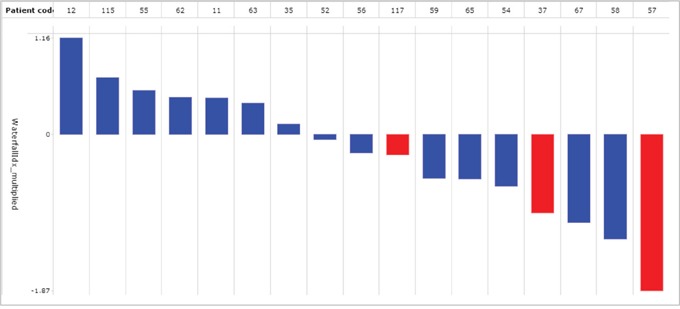
Application of the 95 peptides PLS-DA class prediction model for TNM stage 1 LuAdCa performed on a set of 17 new samples Among the 17 samples, we obtained a proper classification accuracy of 10/17 (59%). In this prediction score chart, samples with a prediction score smaller than zero were allocated to the short-term survivors (red coded) or to the long-term survivors (blue coded) when the prediction performance values were larger than zero. A prediction score situated further away from the decision boundary set at 0 was less likely to really belong to the opposite group.

We detected specific peptide tyrosine phosphorylation in protein lysates obtained from the same-patient lung non-neoplastic versus adenocarcinoma tissues. We assayed adenocarcinoma tissues in either the presence or absence of gefitinib and inhibition profiles were obtained using the “_inh_S” values as described in the Material and Methods section. For the training cohort, a total of 95 out of the 144 peptides available were selected and were present in at least 70% of the LuAdCa resection specimens analysed (see Material and Methods). The “_inh_S” values of the 95 peptides were combined with the patient survivor status in the form of a heatmap (Figure 1). This visualisation showed a clear trend for kinomes of long-term survivors to be more strongly impacted by gefitinib inhibition than that of short-term survivors. A significant difference between the long-term and short-term survivors was characterised for 46 peptides (Figure 1, two-sample two-tailed Student’s t-test O, *p* < 0.05; FDR 10%). Supervised classification performance was examined using partial least squares discriminant analysis (PLS-DA) based on the 95 peptide inhibition profiles of the LuAdCa resection specimens. The prediction accuracy was estimated using leave-one-out cross-validation (LOOCV), and we obtained an accuracy of 14/20 (70%) correctly segregated patients as short- or long-term survivors (Supplementary Figure 1). In addition, we applied this supervised classification model to our validation cohort of patients. Of the 17 patients in the validation cohort, we obtained an accuracy of 10/17 (58.8%) of patients correctly classified regarding their long- versus short-term survival status (Figure 2).

By applying gefitinib and the experimental approach described above to TNM stage 2 protein lysates, a similar analysis did not detect any differences between 5 long- and 5 short-term survivors (data not shown).

### A same-patient comparison of intrinsic kinase activities present in LuAdCa versus lung non-neoplastic tissues did not predict survival

In all LuAdCa protein lysates tested, we noticed a conspicuous and TNM stage-independent tyrosine phosphorylation of substrates. Lower levels of kinase substrate phosphorylation were detected for protein lysates of non-neoplastic tissues. Nonetheless, despite large differences in substrate phosphotyrosine levels, the calculated “_nor_S” value in both supervised and unsupervised clustering analysis produced no correlation of substrate phosphorylation patterns with known clinical parameters (TNM stage, tumour size, histomorphologic grade, anatomical location of the tumour, smoking status, age and gender) involved in lung cancer aetiology (data not shown). In particular, the patient survival status was not correlated with phosphorylation of the 95 selected peptides (Supplementary Figure 2). For the “_nor_S” ratio, a paired two-tailed Student’s t-test only detected 6 peptides (ERBB4_1181_1193; B3AT_39_51; PGFRB_1014_1028; ERBB2_870_882; RBL2_99_111; EGFR_1165_1177) out of the 95 peptides with significant differences between long- and short-term survivors (*p* < 0.05, FDR = 80%). In addition, the supervised PLS-DA classification analysis did not discriminate between long- versus short-term survivors.

The same analysis of “_nor_S” ratio for LuAdCa TNM stage 2 also did not yield any useful classification model. Other clinical parameters (TNM stage, tumour size, histomorphologic grade or anatomical location of tumour, smoking status, age and gender) were not correlated with any characteristic substrate tyrosine phosphorylation patterns (data not shown).

### Estimate of a correction parameter for optimal cutting temperature medium-induced differences in the inhibition of peptide substrate phosphorylation

We noticed a reduced effect of added gefitinib during the phosphorylation inhibition assay in recently collected malignant and non-neoplastic protein lysates. Moreover, this observation seemed to appear in parallel with the presence of a whitish background on images obtained from the top surface of the ceramic microarrays. We searched for causes explaining such an effect and ruled out the implication of either short versus long storage time after protein sample extraction or of heterogeneous batch processing of protein lysates (data not shown). Instead, we documented that the clear reduction in overall phosphorylation levels coincided with the year 2007 and the introduction of optimal cutting temperature (OCT) medium for improved embedding of resection specimens (Supplementary Figure 3). To correct the data for the OCT medium confounding effect, a median centring was performed on the “inhS” values of each peptide, separately for with or without OCT samples. Supplementary Figure 4 illustrates the OCT corrective estimate “inhScor” on the signals of the PLCG1 (764-778) peptides. All the 144 peptides were similarly corrected.

### Selected tyrosyl-phosphorylated substrates involved in FER/FES and JAK/STAT pathways predict the survival status of long- versus short-term TNM stage 1 LuAdCa patients

**Figure 3:**
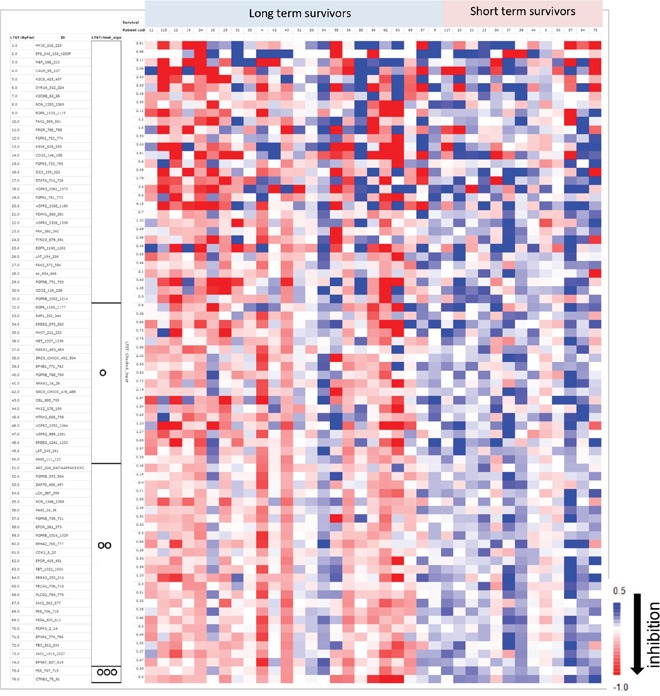
Colour map representation of gefitinib-induced tyrosine phophorylation inhibition in peptide profiles obtained with the combined 37 patients described in Figures 1 and 2 Patient samples are sorted in columns according to their survival status. Rows represent the peptides sorted according to their correlation with survival status. As depicted in the colour bar scale, a red colour indicates a relatively high “_inh_S_cor_” value, a Log2-transformed ratio of tyrosine phosphorylation inhibition by gefitinib. The significance obtained in the *t* test per peptide is indicated on the left side of the figure using the following coding: OOO, *p* < 0.001; OO, *p* < 0.01; O, *p* < 0.05; *p* ≥ 0.05 otherwise.

**Figure 4:**
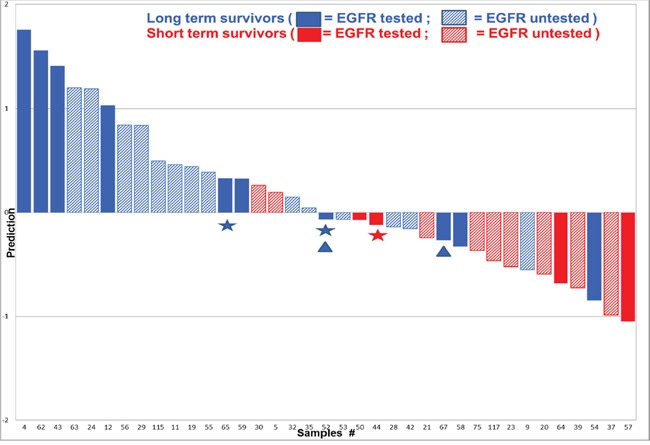
PLS-DA class prediction for TNM stage 1 LuAdCa performed with the 76 selected peptides Predictive performance was examined using PLS-DA and 10-fold cross-validation. Of the combined 37 patients described in Figures 1 and 2, we obtained an accuracy of 27/37 (73%) of proper classification. The predicted class is indicated by the prediction score (y-axis), where a prediction score > 0 indicates a long-term survivor, and prediction score < 0 indicates a short-term survivor. The known class is indicated by plain or dashed blue-coded bars for long-term and plain or dashed red-coded bars for the short term survivors. Prediction performance, which is situated further away from the decision boundary set at 0, is less likely to belong to the opposite group. We randomly tested 13 patients (plain colour bars) for *EGFR* mutations, and 11 of them for *EGFR* amplification (patients #4 and #43 were not tested). We found that 30% (4/11) of the LuAdCa resection specimens had either mutated (triangle) and/or amplified *EGFR* (star).

With the corrective estimate “_inh_S_cor_” applied to all our data, we pooled the TNM stage 1 patient training (Figure 1) and validation cohorts (Figure 2) and re-analysed the data. In this pooled cohort, we selected 76 peptides with a clear phase of exponential growth during the kinase assay in at least 70% of the analysed samples. The “_inh_S_cor_” ratio was represented for each of the 76 peptide phosphotyrosines over the survivor status of the patient in the form of a heatmap. In Figure 3, the peptides are sorted according to the correlation of “_inh_S_cor_” with survival status. Most discriminative peptides were sorted at the bottom of the heatmap and exhibited a relatively higher inhibition level in the long- versus short-term survivors (Figure 3, two-sample two-tailed Student’s t-test OOO, *p* < 0.001; OO, *p* < 0.01). Table 2 also lists the 26 tyrosine kinase peptide substrates with significantly higher inhibition in protein lysates of long- versus short-term survivors (two-sample two-tailed Student’s t-test *p* < 0.01, FDR < 3%). Among the 26 peptide substrates, members of the JAK/STAT- (EPOR, JAK1, JAK2, FES) the FER/FES- (FER, FES, CTNNB1), and PDGFR cytoskeleton remodelling (PDGFRB, PIK3RI, PAXI, PLCG1) but also EphrinA (EPHA1, EPHA2, EPHA7, LCK) (Supplementary Figure 5) signalling pathways were identified. The upstream kinase analysis putatively suggested changed in the activity of SRC family kinases, including SRC, HCK, LCK consistent with the signalling pathways associated with the 26 peptide substrates (upstream kinase analysis data not shown). Supervised classification analysis was performed using PLS-DA based on the 76 peptide inhibition profiles. Prediction accuracy was estimated using 10-fold cross-validation, and 73% of the patients were correctly classified as short- or long-term survivors (Figure 4).

**Table 2:** List of 26 peptide substrates identified after gefitinib-induced tyrosine phophorylation inhibition which are significantly differentially affected in kinomes of long- versus short-term TNM stage I lung adenocarcinoma patient survivors

Position of peptide	Sequence	Tyr site	Uniprot	Common name	P value
ART_004_EAIYAAPFAKKKXC	EAIYAAPFAKKK	NA	NA	Artificial peptide substrate	< 0.01
CDK2_8_20	EKIGEGTYGVVYK	[15, 19]	P24941	Cell division protein kinase 2	< 0.01
CTNB1_79_91	VADIDGQYAMTRA	[86]	P35222	Catenin beta-1 (Beta-catenin).	< 0.01
EPHA1_774_786	LDDFDGTYETQGG	[781]	P21709	Ephrin type-A receptor 1 precursor	< 0.01
EPHA2_765_777	EDDPEATYTTSGG	[772]	P29317	Ephrin type-A receptor 2 precursor	< 0.01
EPHA7_607_619	TYIDPETYEDPNR	[608, 614]	Q15375	Ephrin type-A receptor 7 precursor	< 0.01
EPOR_361_373	SEHAQDTYLVLDK	[368]	P19235	Erythropoietin receptor precursor (EPO-R).	< 0.01
EPOR_419_431	ASAASFEYTILDP	[426]	P19235	Erythropoietin receptor precursor (EPO-R).	< 0.01
FER_707_719	RQEDGGVYSSSGL	[714]	P16591	Proto-oncogene tyrosine-protein kinase FER	< 0.01
FES_706_718	REEADGVYAASGG	[713]	P07332	Proto-oncogene tyrosine-protein kinase Fes/Fps	< 0.01
JAK1_1015_1027	AIETDKEYYTVKD	[1022, 1023]	P23458	Tyrosine-protein kinase JAK1	< 0.01
JAK2_563_577	VRREVGDYGQLHETE	[570]	O60674	Tyrosine-protein kinase JAK2	< 0.01
LCK_387_399	RLIEDNEYTAREG	[394]	P06239	Proto-oncogene tyrosine-protein kinase LCK	< 0.01
P85A_600_612	NENTEDQYSLVED	[607]	P27986	Phosphatidylinositol 3-kinase regulatory subunit alpha	< 0.01
PAXI_24_36	FLSEETPYSYPTG	[31, 33]	P49023	Paxillin.	< 0.01
PDPK1_2_14	ARTTSQLYDAVPI	[9]	O15530	3-phosphoinositide-dependent protein kinase 1	< 0.01
PECA1_706_718	KKDTETVYSEVRK	[713]	P16284	Platelet endothelial cell adhesion molecule precursor (PECAM-1)	< 0.01
PGFRB_1002_10	LDTSSVLYTAVQP	[1009]	P09619	Beta-type platelet-derived growth factor receptor precursor	< 0.01
PGFRB_572_584	VSSDGHEYIYVDP	[579, 581]	P09619	Beta-type platelet-derived growth factor receptor precursor	< 0.01
PGFRB_709_721	RPPSAELYSNALP	[716]	P09619	Beta-type platelet-derived growth factor receptor precursor	< 0.01
PLCG1_764_776	IGTAEPDYGALYE	[771, 775]	P19174	1-phosphatidylinositol-4, 5-bisphosphate phosphodiesterase gamma-1	< 0.01
PRRX2_202_214	WTASSPYSTVPPY	[208, 214]	Q99811	Paired mesoderm homeobox protein 2 (PRX-2)	< 0.01
RET_1022_1034	TPSDSLIYDDGLS	[1029]	P07949	Proto-oncogene tyrosine-protein kinase receptor ret precursor	< 0.01
RON_1346_1358	SALLGDHYVQLPA	[1353]	Q04912	Macrophage-stimulating protein receptor precursor	< 0.01
TEC_512_524	RYFLDDQYTSSSG	[513, 519]	P42680	Tyrosine-protein kinase Tec	< 0.01
ZAP70_485_497	ALGADDSYYTARS	[492, 493]	P43403	Tyrosine-protein kinase ZAP-70	< 0.01

### No significant association between patient survival and the epidermal growth factor receptor (*EGFR*) mutation and/or amplification status were observed

From the pooled cohorts with 37 patients, we also randomly assessed 13 LuAdCa resection specimens to determine the *EGFR* status (*EGFR* wild type/mutant and/or *EGFR* amplification), an important parameter that is used to help guide treatment decisions. As indicated in Figure 4, we obtained a total rate of 30% of patients with either mutated *EGFR* or with amplified *EGFR*, in comparison to 70% who had the wild type form. The LuAdCa resection specimens with *EGFR* mutation/amplification were equally distributed between long- and short-term survivors.

## DISCUSSION

Protein phosphorylation represents an important and ubiquitous post-translational modification in eukaryotic biological systems and has a prominent role in cancer initiation and progression. The main role of kinases is to turn cellular processes “on” and “off”, and thus the kinome has been the focus of large efforts to understand cancer-modified signalling pathways [18]. In this project, we screened tyrosine kinase activities in 71 freshly frozen primary stage 1 and 2 LuAdCa resection specimen lysates with and without the presence of the PTKI gefitinib. We present the feasibility of discriminating long- versus short-term stage 1 LuAdCa survivors based on tyrosine kinase activities.

Using our approach, sets of activated versus inactivated (or gefitinib bound) protein tyrosine kinases present in the adenocarcinoma lysates were subjected to protein tyrosine kinase activity profiling on the PamChip®4 microarrays. We obtained inhibition profiles that were analysed for representative tumour signatures. Here, gefitinib was used as a tool to increase the discriminative power of the measurements by comparing activities with and without added inhibitor during the assay. The advantages of using PTKI inhibition over direct comparison with a non-neoplastic profile are both technical and biological. From a technical standpoint, quantitation and processing of the same malignant protein lysate, with or without presence of added PTKI, and from the exact same LuAdCa resection specimen tissue provides an advantage. This malignant sample can be measured either with PTKI or without PTKI as a control, thereby reducing differences in baseline signal between patients. From a biological perspective, the advantage lies in the ability to use specific inhibitors that may be efficient against a known signalling pathway important for tumour progression or treatment response. Moreover, drug targeting of a specific signalling pathway may help to better discriminate between sample phenotypes. In our study, gefitinib was selected because it is already used clinically as a treatment for distinct LuAdCa phenotypes [25]. As appears to be the case in our study, gefitinib-induced inhibition of EGFR activity in comparison to overall gefitinib-free tyrosine kinase activity better discriminated LuAdCa resection specimen lysates than direct comparison with non-neoplastic samples.

In TNM stage 1 LuAdCa, the tyrosine kinases responded more strongly to the inhibitory effect of gefitinib in long-term survivors compared with short-term survivors. Moreover, we used a set of 76 peptide inhibition profiles as input for PLS-DA supervised classification analysis, which resulted in correct prediction of survival status for 73% of the patients (based on a 10-fold cross-validation). Interestingly, we detected 26 peptide substrates that were significantly more inhibited in the protein lysates of the long- versus short-term survivors. Pathway analysis may provide hypotheses for further research and follow-up work. Among the 26 peptide substrates that significantly discriminated short- versus long-term survivors, we listed members of the EphrinA-signalling pathway (EPHA1, EPHA2, EPHA7, LCK) [26] and the PDGFR cytoskeleton remodelling pathway (PDGFRB, PIK3RI, PAXI, PLCG1) [27]. Interestingly, expression of PDGFRB in carcinomas is generally restricted to stromal cells of mesenchymal origin and is generally absent in epithelial tumour cells [28]. However, epithelial-mesenchymal transition (EMT) almost universally upregulates PDGFRB expression, and mesenchymal-like NSCLC cells exhibit aberrant PDGFR and FGFR expression. This finding implicates EMT as a mechanism for kinase switching, thereby decreasing cellular sensitivity to EGFR inhibition [27]. We also listed members of the FER/FES (FER, FES, CTNNB1) [29] and the JAK/STAT (EPOR, JAK1, JAK2, FES) pathways [30]. JAK/STAT pathway activation contributes to the acquisition of properties required for tumour invasion and metastasis. STAT3 has a global role in the adaptation of tumour cells to a hypoxic microenvironment, and constitutively active STAT3 leads to increased VEGF expression and increased vasculogenesis [31]. Many of the identified peptide substrates, such as the FER/FES kinases, are involved in one specific task such as cell adhesion, an important process in cancer metastasis. We also report cross-activated kinases, such as JAK’s, that may not only be associated with the EPOR pathway but also be active in several pathways such as with the PDGFRB and EPHA receptor signalling pathways.

A random screen of the LuAdCa resection specimens revealed that 30% of the total specimens analysed had a mutant or amplified *EGFR*, which is similar to the prevalence observed in a randomly selected Caucasian population affected by LuAdCa [32]. To refine our LuAdCa signature, it would still be advantageous to perform a larger screen for the *EGFR* mutant or amplified status in all LuAdCa resection specimens and to analyse known frequent LuAdCa-specific mutations [33].

When we applied the training model to the validation set, we identified assay interferences due to the presence of OCT in the samples embedded after 2007 (which were also overrepresented in the validation set), corresponding to the exact time we implemented our OCT-based embedding procedure to enhance the protection of our resection specimens [34]. Nonetheless, we observed a clear discrimination of long- and short-term survivors in the OCT and non-OCT groups, but with lower overall kinase inhibitions in the LuAdCa resection specimens embedded in OCT. We assume that the “_inh_S_cor_” correction is unnecessary for future studies if a homogeneous type of embedding procedure is used for all the analysed samples. Clearly, the method should also be tested using a large validation set to overcome the effect of any optimistic biases in the current analysis. We were unable to perform such a new blinded analysis due to both the re-analysis of known samples after the application of the OCT corrective estimate and the scarcity of new untested LuAdCa resection specimens. A possible refinement could be achieved with an international validation study encompassing different human ethnic groups. *In silico* human genome analysis has described more than 500 types of DNA signatures with a kinase domain [16]. Ninety are unique tyrosine kinase genes [17] and, as key regulators of cell functions, are directly involved in numerous signal transduction cascades [18]. The ability to use 144 investigator-selected peptides as substrates to extrapolate the overall kinase selectivity of a complex disease such as LuAdCa is limited. In studies assessing kinase selectivity, a larger set of peptide substrates is still recommended [35].

Nevertheless, we observed interesting and potentially clinically applicable differences in the kinomes of long- versus short-term survivors. Currently, molecularly targeted therapies based on small molecules and monoclonal antibodies directed against tyrosine kinases are already approved for lung cancer treatment. Approximately 20% of lung cancer patients can actually be stratified for treatment with EGFR targeting therapies that successfully suppress cell growth and promote cell death. New strategies are further needed to elucidate novel target signalling pathways among the numerous DNA signatures with a kinase domain described *in silico* in the human genome and potentially involved in either lung cancer disease or the lung cancer drug-resistant state [33]. Kinase activities have been used to predict drug and radiation treatment responses for rectal cancer specimens [36]. Our results show that kinomic profiling data are a promising approach in lung cancer as well, that may potentially provide therapeutic drug response information prior to lung cancer patient therapy. Recently, Anderson *et al* [37] developed an interesting approach based on electromagnetic navigational bronchoscopy (ENB) [38] derived lung tumour specimens and kinomic data for “biologically-interrogated’’ ENB specimens using small molecule inhibitors.

Our classifier achieved a rather high positive predictive value since a large proportion of the patients predicted to be long-term survivors were actually long-term survivors (approximately 88% of the long-term survivors, Figure 4). Among the short-term survivors approximately half were classified correctly (57%). With future refinement of our classifier, we would suggest that a patient LuAdCa resection specimen classified as short-term survivors may receive either adjuvant radiotherapy and/or chemotherapy after surgery or an enhanced monitoring by regular CT scan. A further biological and mechanistic interpretation of the observed differences in the 26 peptide substrates that were significantly inhibited in the protein lysates of the long-term survivors presented in this study is still of interest.

## MATERIALS AND METHODS

### Management of collected LuAdCa resection specimens and clinical data from patients

This study was performed in accordance with the Helsinki Declaration and was examined and approved by the Institutional Ethical Review Board of the University Hospital Zürich (UHZ). In January 2003, the UHZ Division of Thoracic Surgery started a collection of same-patient fresh frozen LuAdCa and non-neoplastic tissues. In this study, all the mandatory informed consent approval forms with all clinical interventions and follow-up treatment decisions from every participating lung cancer patient were archived. All lung resection specimens collected until December 2006 were directly snap-frozen in liquid nitrogen and stored at −80°C. As of January 2007, this procedure was updated, and up to the censoring day of January 1, 2013, our lung resection specimens were processed in the following way: i) specimens were embedded in OCT (Tissue-Tek, Miles Inc., Elkhart, IN, USA), and the European standard for tumour banking was applied [34] ii) all our specimens were cryopreserved in the gas phase of a liquid nitrogen tank at −196°C.

### *EGFR* mutational analysis of LuAdCa resection specimens

A set of LuAdCa resection specimens were randomly tested for *EGFR* mutation and amplification status as previously described [39]. To assess *EGFR* overexpression and gene amplification, we performed *CEP7/EGFR* dual colour FISH (Vysis, Abbott AG, Baar, Switzerland) with the Spectrum Orange probe specific for the *EGFR* locus (7p12) and the Spectrum green probe specific for the chromosome 7 centromere (7p11.1 to q11.1). A total of 50-100 non-overlapping tumour cell nuclei were counted. For exon 18 to 21 *EGFR* mutations, tissue areas from FFPE tissue were excised with a puncher and DNA was extracted and amplified by PCR. Finally, the PCR product were analysed by 2% agarose gel electrophoresis and subsequently purified and sequenced.

### Histopathological examination of the specimens and study design

We collected resection specimens with clinical characteristics of LuAdCa TNM stage 1 (1A and 1B) or 2 (2A or 2B) up to 12/2010 (see Supplementary Table 1). Starting with 118 computed database entries we excluded from the study, 13 patients died in manner not attributable to lung cancer (group 1), 10 patients had undergone neoadjuvant chemotherapies (group 2), 5 patients had either lost or unusable specimens (group 3) and 2 patients remained unreachable for follow-up (group 4). At the time of surgical resection, we conducted histopathological examination and tumour staging according to the 6^th^ TNM classification of malignant lung tumours [40] and estimated the grade of tumour differentiation [41]. All the LuAdCa resection specimens included in this study were mounted in OCT and cryosectioned in eight-micron thick sections with an HM 560 cryostat (Microm) set at −25°C and further stained with haematoxylin and eosin. We excluded 7 patient specimens with less than the estimated 30% minimal area percentage of the LuAdCa resection specimen occupied by tumour cells (group 5) and 6 patient specimens with areas of desiccated, necrotic, or mixed NSCLC phenotypes (group 6). The collected resection specimens had a median tumour cell content of 100% with a range of 30%-100% and an average tumour cell content of 88.3%.

Finally, after thorough inspection of peptide tyrosine phosphorylation kinetics, the assay results for 4 patients were removed because two of our predefined inclusion criteria were unmet: 1) extensive image saturation as a result of either very low or highly saturated peptide tyrosine phosphorylation; 2) data points in the principal component analysis (PCA) that were clear outliers from the rest of the dataset (group 7).

The patient DSS time was computed either as the months elapsed from the day of surgery until death as a result of a relapse of their lung cancer or, for patients above our assigned cut-off values, as the total number of months elapsed until the last available follow-up. Briefly, we collected 83 TNM stage 1 (33 with 1A; 50 with 1B) and 35 TNM stage 2 (11 with 2A; 24 with 2B) LuAdCa resection specimens. We also had to allocate patients into training and validation cohorts, and we selected a 1 to 0.85 ratio for the stage 1 and a 1 to 0.7 ratio for the stage 2. We constructed both the TNM stage 1 and 2 training cohorts with the oldest resection specimens in our tumour collection. Data from the 6^th^ edition textbook of the TNM classification stated a 5-year survival rate ranging from 73% for stage 1A to 52% for stage 1B and of 48% for stage 2A to 30% for stage 2B [42].

To distinguish short- versus long-term survivors and because of the incomplete 5-year follow-up time in our study population, we assigned shorter cut-off values in this study. In summary, and taking into account the high proportion of TNM B versus TNM A stages, the balancing of the short- versus long-term survivors training cohorts, the allocation in training and validation cohorts and the actual survival value, we selected 62% of our stage 1 and 45% of our stage 2 patient populations as cut-off values for survival. We then deduced the months cut-off value corresponding to the two above-mentioned survival percentages. For the 118 selected patients, the long- versus short-term survivor cut-off values were set at 53 months for stage 1 and 23.5 months for stage 2.

### Preparation of LuAdCa resection specimens for the *ex vivo* kinase assay

Two cryosections with a thickness of 30 microns and covering an area of approximately 5×5 mm (or 1-1.5 mm^3^ of tissue) were collected from each of the LuAdCa and same-patient non-neoplastic resection specimens. Prior to protein extraction, the OCT-embedded cryosections were quickly thawed and briefly pelleted at 3000 rpm for 0.5 min in a microcentrifuge set at 4°C. The protein lysates were extracted and resuspended 30 times on ice using wide bore pipet tips in 100 μl of ice-cold Pierce mammalian extraction buffer (M-PER Thermo Fisher Pierce, Rockford, IL, USA) containing both Pierce Halt Phosphatase Inhibitor Cocktail and Pierce Halt Protease Inhibitor Cocktail EDTA free (Thermo Fisher Pierce, Rockford, IL, USA). All samples were pelleted at 3000 rpm for 0.5 min in a microcentrifuge set at 4°C, processed again 30 times using wide bore pipet tips and finally lysed for 15 mins on ice. After centrifugation at 10000 rpm for 15 mins and 4°C, each protein lysate supernatant was aliquoted into 10 tubes and quickly frozen before storage at −80°C. The protein concentration was estimated with the Pierce micro BCA kit (Thermo Fisher Pierce, Rockford, IL, USA) using bovine serum albumin (BSA) as a standard. Additionally, malignant samples were also analysed with a 10 μM final concentration of gefitinib (CAS 184475-35-2, Cayman Biochemicals, Ann Arbor, MI). The 40 μl final volume of kinase master mix containing the kinase assay buffer (50 mM Tris-HCl pH 7.5, 10 mM MgCl2, 1mM EGTA, 2 mM dithiothreitol, 0.01% Brij 35, 1 mg/ml BSA, and 12.5 μg/ml FITC-labelled PY-20 antibody) was prepared according to the instructions provided by the manufacturer (PamGene, 's-Hertogenbosch, The Netherlands) and assayed with the following modifications: i) we tested 5 μg of the extracted protein lysate in a 1 to 5 μl final volume of M-Per lysis buffer and, ii) we included either 2 μl of a 200 μM stock of gefitinib in DMSO or 2 μl DMSO. Finally, to synchronise the twelve kinase reactions, we added 4 μl of a 4 mM ATP stock solution to reach a 0.4 mM final ATP concentration.

### Multiplex profiling of protein tyrosine kinase substrates

Multiplexed *ex vivo* profiling of protein tyrosine kinase substrates from human LuAdCa protein lysates was performed. As substrates, we used a PTK PamChip®4 microarrays (PamGene, 's-Hertogenbosch, The Netherlands) dotted with 13-15 amino acid consensus tyrosine phosphorylation peptide sequences that were identified in both the SwissProt and PhosphoBase databases [35]. The list of 144 different peptide substrates immobilised through 2 supplementary amino acid residues to the porous ceramic microarray is provided in Supplementary Table 2. Substrate tyrosine phosphorylation was detected using fluorescein-labelled anti-phosphotyrosine antibodies (clone PY-20-FITC). The kinase reactions were performed with up to 12 arrays in parallel on a PamStation®12 (PamGene, 's-Hertogenbosch, The Netherlands) at 30°C. The time course of phosphorylation was followed for one hour with 92 pumping cycles through the porous ceramic microarray by recording a fluorescence image of each array every fifth pump cycle with a CCD camera. A randomisation scheme for duplicate measurements of all samples was applied as follows: two distinct runs of the PamStation®12 were performed on two independent PTK PamChip®4 microarrays with two freshly thawed protein lysate aliquots.

### Image filtering, data adaptation and statistical analysis

Duplicate multiplex kinomic profiles of same-patient LuAdCa or non-neoplastic resection specimens were obtained. The software package BioNavigatoR (version 5.2 - 6.2; PamGene, 's-Hertogenbosch, The Netherlands) was used for inspection of the recorded images for quality control investigations and for quantitation of the tyrosine phosphorylation signals. This process included standard procedures for the automatic edge detection of microarrayed spots. For each spot, the tyrosine phosphorylation signal was used as the median spot pixel value minus the median local background pixel value. Measurements were carefully inspected and repeated for one or both replicates in the case of evident mechanical or technical problems during data acquisition, and for replicates with measurement inconsistencies. For further analysis, the signal obtained after a final washing step at the end of the incubation was used. This signal was recorded using 3 different CCD camera exposure times. A standard procedure was applied to combine these readings into a single signal value with a maximum dynamic range. Only peptides with clear growth of the tyrosine phosphorylation signal during the assay for at least 70% of non-neoplastic and malignant samples (without gefitinib) were selected for further analysis.

The ratio of peptide phosphorylation “_nor_S” between same-patient non-neoplastic and malignant resection specimens was calculated as the Log2 ratio of the signal obtained for the malignant (Sm) or non-neoplastic (Snn) resection specimens: _nor_S = Log2 (Sm/Snn)

The inhibition value “_inh_S” was calculated as the Log2 ratio of the peptide phosphorylation signal obtained with (Si) or without (Sm) addition of gefitinib in malignant resection specimen lysates: _inh_S = Log2 (Si/Sm)

First, a Log2 ratio per run was calculated, and duplicated ratios were subsequently averaged. We performed a comparative analysis of ratios from between two groups of samples (e.g., long-term versus short-term survivors), which included heatmap visualisation of the data and per-peptide two-sample two-tailed Student’s t-tests to identify peptide that were significantly different between the groups.

Unsupervised multivariate clustering of samples was inspected in low-dimensional score plots of the measurements obtained by PCA (see Supplementary Figure 6). Classification analysis was performed using PLS-DA (see also Supplementary Text 1, Supplementary Figure 7 [43]), a method that has been previously found to perform well with PamChip kinomic data [36, 44]. This analytical approach results in a prediction score that can be used to predict the class of new samples. The predictive performance of the classification model was estimated with LOOCV or 10-fold cross-validation or by applying the classification model to an independent validation set.

During the course of the analysis, we realised that the OCT embedding medium used to store lung resection specimens had an effect on both the “_nor_S” and “_inh_S” values. We designed and applied a correction in which median centring was performed on the “_inh_S” values of each peptide, separately for the samples with or without OCT. We obtained corrected values named “_nor_S_cor_” and “_inh_S_cor_”. Statistical analyses were performed using BioNavigatoR (versions 5.2 -6.2, PamGene, 's-Hertogenbosch, The Netherlands) interfaced with R (versions 2.15.3 [45]) and MATLAB R2010b, (Mathworks Inc., Natick, MA, USA).

## SUPPLEMENTARY MATERIALS FIGURES AND TABLES







## Abbreviations

DSS: Disease-specific survival
EGFR: Epidermal growth factor receptor
ECN: Electromagnetic navigational bronchoscopy
LOOCV: Leave-one-out cross-validation
LuAdCa: Lung adenocarcinoma
NSCLC: Non-small cell lung cancer
OCT: Optimal cutting temperature medium
PCA: Principal component analysis
PLS-DA: Partial least squares discriminant analysis
PTKI: Protein tyrosine kinase inhibitor
TNM: A cancer staging system describing tumour size (T), lymph node involvement (N) and distant metastasis (M).
