# Supplementary material for: Ex vivo multiplex profiling of protein tyrosine kinase activities in early stages of human lung adenocarcinoma
Source: Oncotarget. 2017 Aug 2;8(40):68599–613. doi: 10.18632/oncotarget.19803 (PMC5620281; doi:10.18632/oncotarget.19803)
Supplement: Supplementary file 1 [file oncotarget-08-68599-s001.pdf]

Ex vivo multiplex profiling of protein tyrosine kinase activities in early stages of human lung adenocarcinoma

SUPPLEMENTARY MATERIALS

Supplementary Text 1: classification analysis using partial least squares discriminant analysis

Partial Least Squares Discriminant Analysis (PLS-DA) is a supervised classification method that has been widely applied to classification problems. The method is related to classical Linear Discriminant Analysis (LDA). However, PLS-DA readily works when the number of variables is larger than the number of observations and may be particularly suited to cases when there is a high degree of collinearity between the predictor variables [1]. PLS-DA usually ranks among the best performing classifiers with PamChip® peptide microarray data and was previously applied to classification problems with clinical samples (e.g. [2, 3]).

In the present study we apply PLS-DA without any explicit selection of discriminative peptides. PamChip® kinase activity arrays contain up to 144 substrate peptides of which typically 80-120 peptides are retained for further analysis by applying an overall signal threshold during the data preprocessing stages. Hence, a subset of peptides is selected (here 95 and 76 peptides, respectively) without consideration of their discrimination between the classes of interest. During training of the PLS-DA classifier all retained variables are then implicitly weighted for their importance in discriminating between the classes. As optimizing a model for predictive performance may not be the best way to identify variables of biological interest [4, 5]), we prefer other forms of comparative analysis as a basis for biological interpretation.

An estimate of the predictive performance (e.g. the correct classification rate) when it is applied to new observations is best obtained by application to a large set of observation of known class, but with the class blinded until after the predictions have been submitted. More often than not this is infeasible, e.g. because the number available observations with known class is too low. In that case, cross-validation (CV) can be used to provide an estimate of predictive performance with new samples. Proper CV will yield an unbiased estimate of prediction performance (note: even in the presence of classifier overfitting). However, care must be taken to make sure that on each iteration of CV the classifier is constructed independent of the observations that are used for testing [6, 7]. Specifically for the present PLS-DA method, an optimal number of PLS components (i.e. the dimension reduction) must be selected. This was done by cross validation of the training set only (double cross-validation, e.g. [8]). Also, per variable mean and standard deviation used for autoscaling of the variables were estimated on the respective training sets only, and applied to the test samples before prediction. In this way, we have applied leave-one-out-cross-validation (LOOCV) to our initial smaller training set and 10-fold CV to the final pooled data set. For the latter, repeated 10-fold cross validation yielded a stable result, in most repeats the performance quoted in the main text was obtained (correct classification 70%, PPV 85%, NPV 55%). For a few repeats, the correct classification rate was somewhat higher. The selected number of PLS components was 1, throughout.

The approximate 2:1 ratio of “long-term survivors” and “short-term survivors” observations in the final pooled set in this study had to be taken into account while training the classifier. This was done by applying a Random Under Sampling (RUS) scheme in which for each training-set a set of 20 sub-classifiers were trained on all the available “short-term survivors” and an equally large random subset of “long-term survivors”. A prediction score for new samples was calculated as the median prediction score of the 20 sub-classifiers. A permutation test was run in which the full 10-fold cross validation procedure was repeated 500 times but with the “long-term survivor” and “short-term survivor” class labels randomly re-assigned to the samples. The empirical cumulative distribution of the correct classification rate that was obtained under this condition of “uninformative data” is shown in Supplementary Figure 7. It may be seen that the cross validation procedure is essentially unbiased because the median correct classification rate under label permutation is near 50% (51%). Also, a correct classification rate ≥ 70% is obtained only in 2-3% of the permuted cases, which shows that the classifier obtained with correct label assignment has a significant prediction performance ($p < 0.05$).

REFERENCES

1. Rayens W, Barker M. Partial least squares for discrimination. J Chemometrics. 2003; 17:166-73.
2. Tahiri A, Røe K, Ree AH, de Wijn R, Risberg K, Busch C, Lønning PE, Kristensen V, Geisler J. Differential inhibition of *ex-vivo* tumor kinase activity by vemurafenib in BRAF(V600E) and BRAF wild-type metastatic malignant melanoma. PLoS One. 2013; 8:e72692.

3. Folkvord S, Flatmark K, Dueland S, de Wijn R, Grøholt KK, Hole KH, Nesland JM, Ruijtenbeek R, Boender PJ, Johansen M, Giercksky KE, Ree AH. Prediction of response to preoperative chemoradiotherapy in rectal cancer by multiplex kinase activity profiling. Int J Radiat Oncol Biol Phys. 2010; 78:555-62.
4. Venet D, Dumont JE, Detours V. Most random gene expression signatures are significantly associated with breast cancer outcome. PLoS Comput Biol. 2011; 7:e1002240.
5. Ein-Dor L, Zuk O, Domany E. Thousands of samples are needed to generate a robust gene list for predicting outcome in cancer. Proc Natl Acad Sci U S A. 2006; 103:5923-8.
6. Boulesteix AL, Strobl C, Augustin T, Daumer M. Evaluating microarray-based classifiers: an overview. Cancer Inform. 2008; 6:77-97.
7. Simon R, Radmacher MD, Dobbin K, McShane LM. Pitfalls in the use of DNA microarray data for diagnostic and prognostic classification. J Natl Cancer Inst. 2003; 95:14-8.
8. Westerhuis JA, Hoefsloot HC, Smit S, Vis DJ, Smilde AK, van Velzen EJ, van Duijnhoven JP, van Dorsten FA. Assessment of PLSDA cross validation. Metabolomics. 2008; 4:81-9.

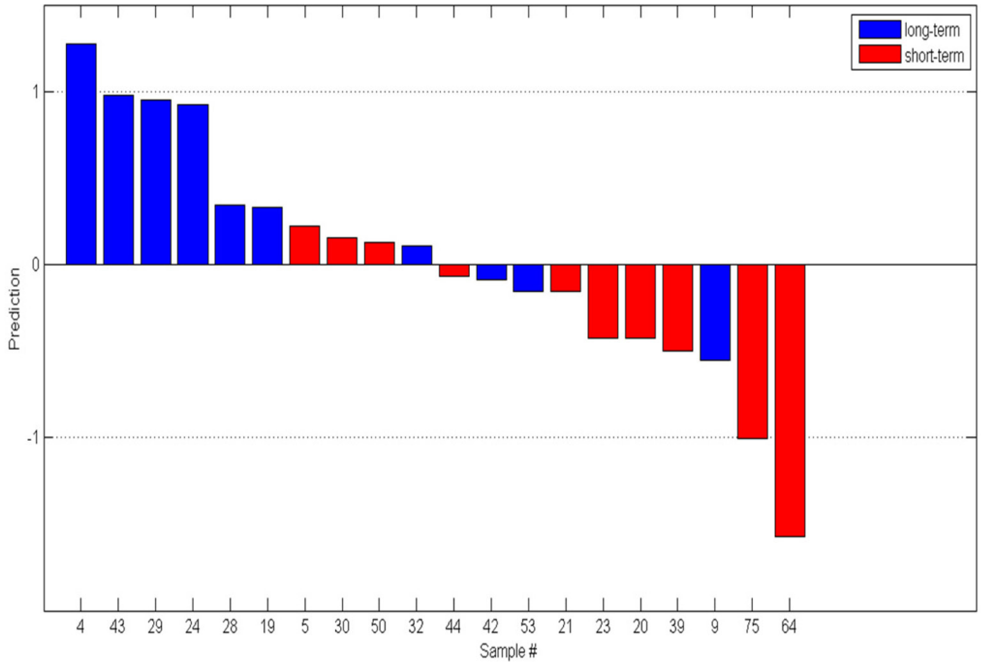

Supplementary Figure 1: Training cohort classification of TNM stage 1 LuAdCa patients in long- versus short-term survivors according to their substrate phosphorylation status in the presence of gefitinib. PLS-DA class prediction for TNM stage 1 LuAdCa patients based on 95 peptides. The prediction score was obtained by LOOCV of the PLS-DA classifier. The rate of correct classification was 70% (or 65-70% by repeated 10-fold cross-validation). In this prediction score chart, samples with a prediction score smaller than zero were allocated to the short-term survivors (red coded) or to the long-term survivors (blue coded) when prediction performance values were larger than zero. A prediction score situated further away from the decision boundary set at 0 was less likely to actually belong to the opposite group.

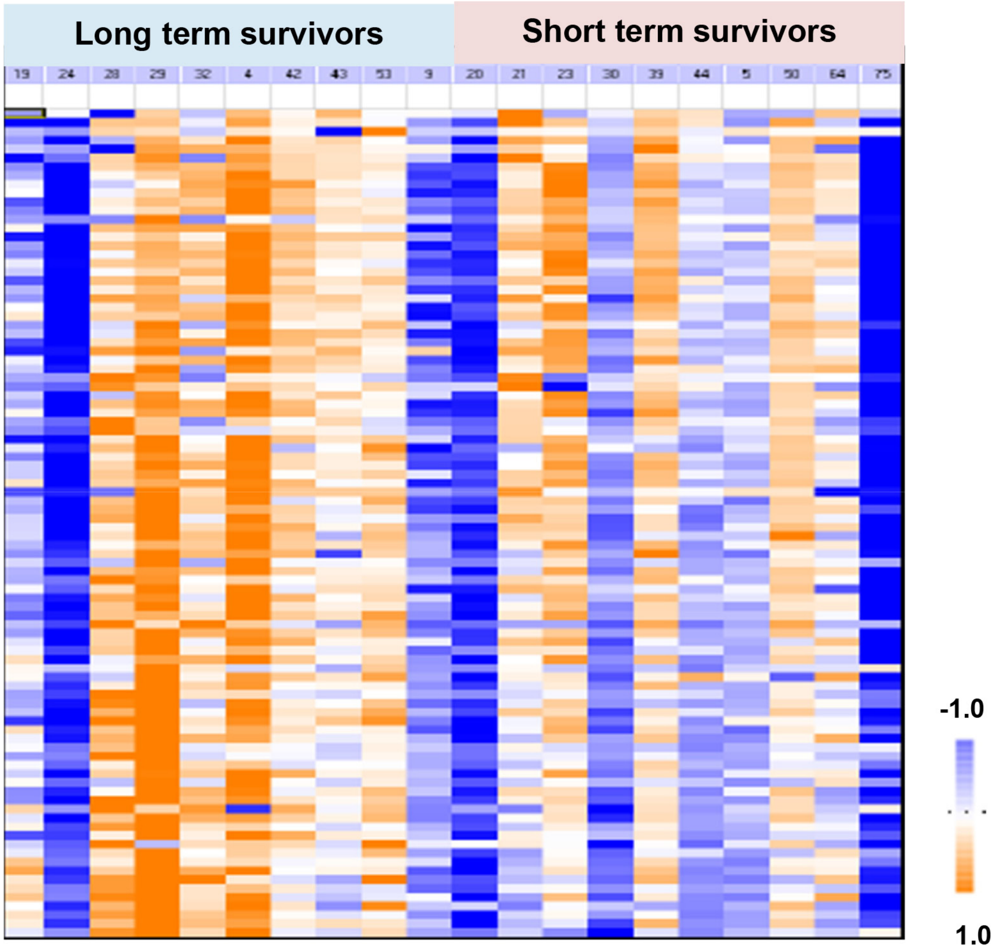

Supplementary Figure 2: Tyrosine phosphorylation of selected peptide substrates in non-neoplastic versus LuAdCa resection specimens. The 95 PCA-selected peptides are represented as "$_{nor}$S" values, a Log2-transformed ratio of tyrosine phosphorylation for non-neoplastic versus TNM stage 1 LuAdCa resection specimens. The patient LuAdCa resection specimens are plotted in a heatmap according to either long- or short-term survivor status. Tyrosine phosphorylation is scaled per peptide, and an orange colour indicates higher phosphorylation in tumour versus non-neoplastic samples.

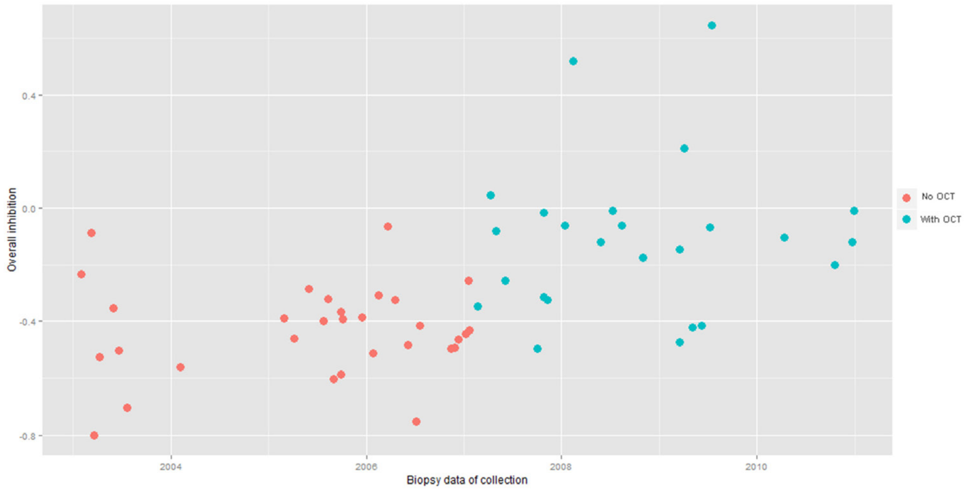

Supplementary Figure 3: Presence or absence of OCT in extracted protein lysates induce differences in inhibition of overall kinase activity. Overall inhibition (y-axis) is plotted as a function of the LuAdCa resection specimen collection time scale (x-axis) from the first collection in January 2003 to the last collection in December 2010. Peptide phosphorylation inhibition decreased over time along the y-axis. LuAdCa resection specimens embedded with OCT are represented by blue circles, whereas specimens without OCT are represented by red circles.

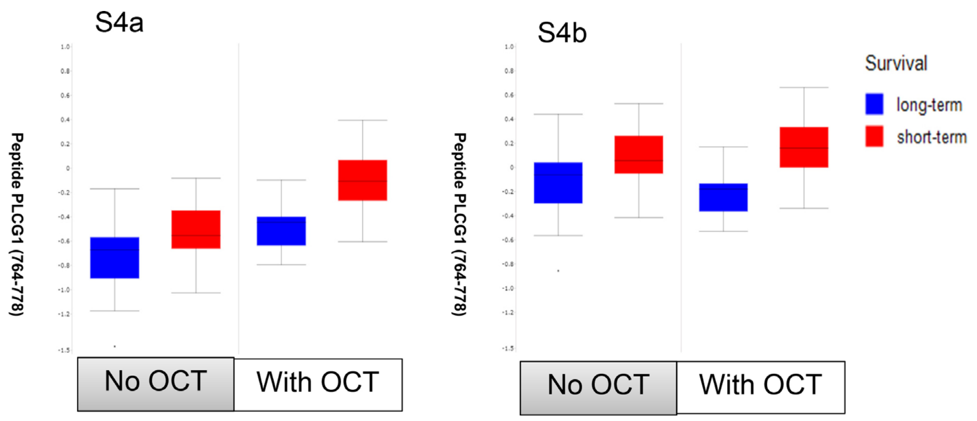

Supplementary Figure 4: Estimate and correction applied for the difference in overall peptide phosphorylation observed in the presence or absence of OCT. OCT was recovered at the time we prepared LuAdCa resection specimen cryosections and accumulated into protein lysates. The whitish OCT component dissolved into the protein lysates was then deposited at each pumping cycle over the time course of the kinase assay into the porous ceramic material of the microarray. S4a: Boxplots of uncorrected data for phosphorylation of a single peptide PLCG1 (764-778) indicate the data for samples embedded in the presence or the absence of OCT. Median centring was performed on the "inhS" values of each peptide, separately for the samples with or without OCT. Median values are represented in the box-blot by a line. A lower degree of inhibition can be noted for the OCT-embedded samples compared with the non- OCT embedded samples, but the inhibition for long-term survivors was higher than that for short-term survivors in both groups. S4b: Typical effect of OCT correction. The data for the two types of sample preparations are on the same scale. Long-term and short-term survivors are presented in blue and red colours, respectively. This estimate was calculated for all the 144 peptides.

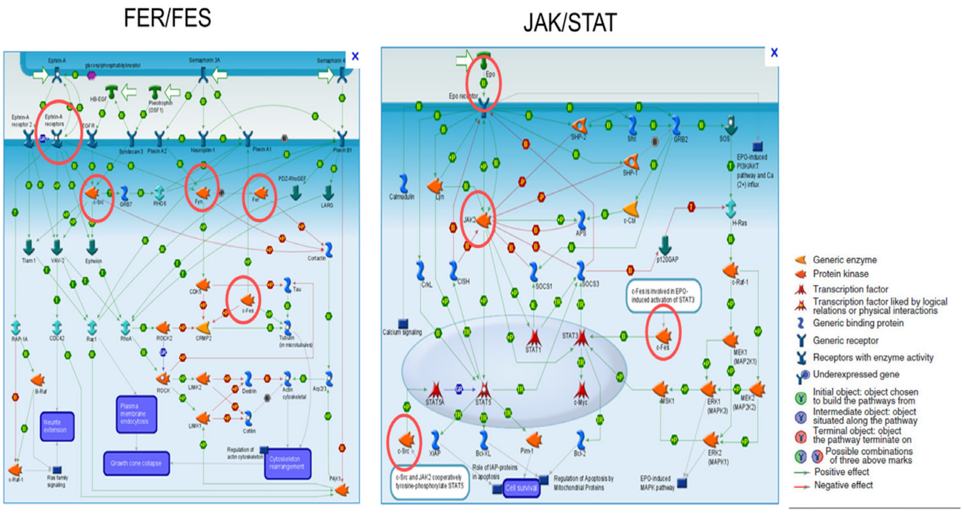

Supplementary Figure 5: Kinases and pathways affected by *ex vivo* exposure to gefitinib. Pathway analysis performed in MetaCore™ (Thomson Reuters) with kinase substrates of long-term survivors identified as mostly affected by gefitinib inhibition in our *ex vivo* kinase assay. The kinases involved in the JAK/STAT (EPOR, JAK1, JAK2, FES) or FER/FES (FER, FES, CTNNB1) pathways are shown in red circles. Unrepresented in this figure are members of the EphrinA signalling pathways (EPHA1, EPHA2, EPHA7, LCK) and of the cytoskeletal remodelling PDGFR pathways (PDGFRB, P85A, PAXI).

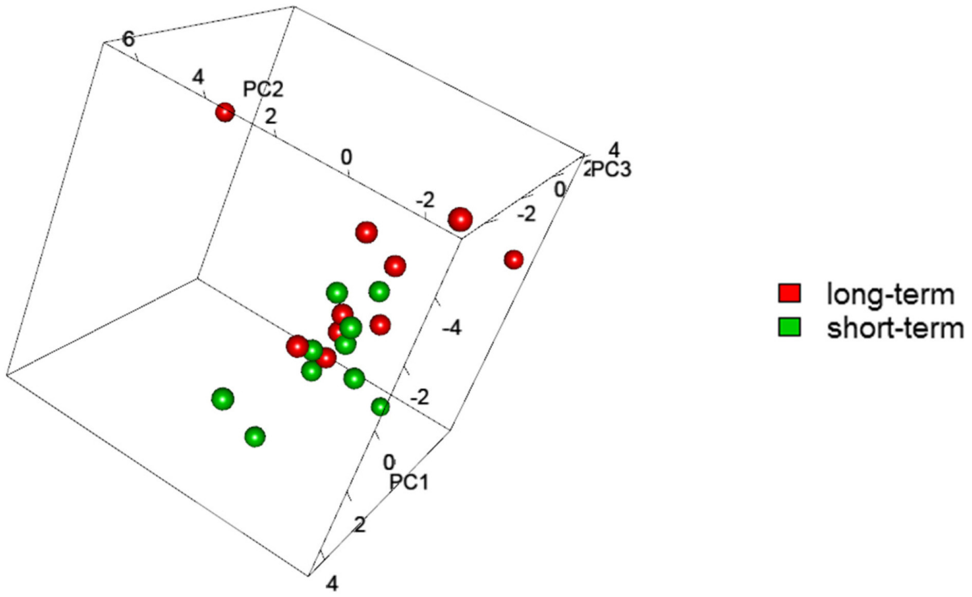

Supplementary Figure 6: Exploratory PCA in the presence of gefitinib. Exploratory PCA for the 20 samples selected to obtain the initial model. Each point represents the PCA score for PC1-3 for each of the patient LuAdCa specimens and is coloured according to the survival status of the corresponding patient.

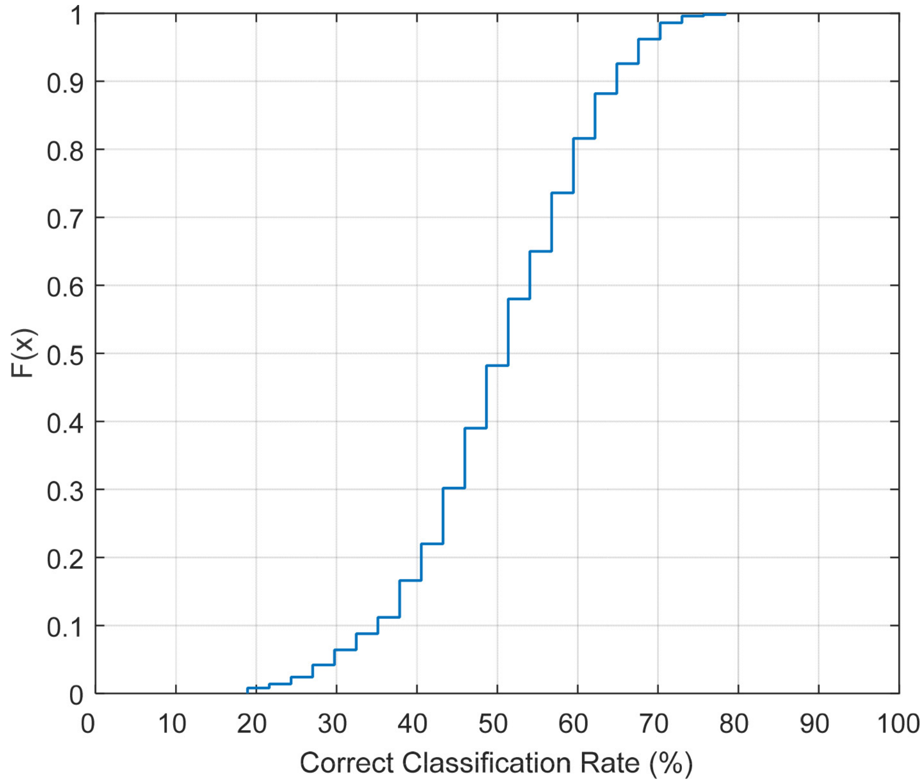

Supplementary Figure 7: Empirical cumulative distribution of the correct classification rate obtained from a permutation test. The analysis shown in Figure 2 was repeated 500 times with random re-assignment of the "long-term survivor" and the "short-term survivor" class label to the observations. The median correct classification rate under permutation was 51%, and the 95% quantile point of the distribution was at 68% with 2-3% of permuted cases resulting in a correct classification rate > 70%.

Supplementary Table 1: Detailed clinical characteristics of the patients

See Supplementary File 1

Supplementary Table 2: List of 144 peptides substrates

See Supplementary File 2
